# International Differences in Treatment and Clinical Outcomes for High Grade Glioma

**DOI:** 10.1371/journal.pone.0129602

**Published:** 2015-06-10

**Authors:** Li-Nien Chien, Quinn T. Ostrom, Haley Gittleman, Jia-Wei Lin, Andrew E. Sloan, Gene H. Barnett, J. Bradley Elder, Christopher McPherson, Ronald Warnick, Yung-Hsiao Chiang, Chieh-Min Lin, Lisa R. Rogers, Hung-Yi Chiou, Jill S. Barnholtz-Sloan

**Affiliations:** 1 School of Health Care Administration, College of Public Health and Nutrition, Taipei Medical University, Taipei, Taiwan; 2 Case Comprehensive Cancer Center, Case Western Reserve University School of Medicine, Cleveland, Ohio, United States of America; 3 Department of Neurosurgery, Taipei Medical University Wan-Fang Hospital, Taipei, Taiwan; 4 Department of Neurosurgery, Taipei Medical University Shuang-Ho Hospital, New Taipei City, Taiwan; 5 Brain Tumor and Neuro-Oncology Center, Department of Neurosurgery, University Hospitals Case Medical Center, Case Western Reserve School of Medicine, Cleveland, Ohio, United States of America; 6 Rose Ella Burkhardt Brain Tumor and Neuro-Oncology Center, Department of Neurosurgery, Neurological Institute, Cleveland Clinic, Cleveland, Ohio, United States of America; 7 Dardinger Neuro-Oncology Center, Department of Neurosurgery, James Comprehensive Cancer Center and The Ohio State University Medical Center, Columbus, Ohio, United States of America; 8 Ph.D. Program for Neural Regenerative Medicine, College of Medical Science and Technology, Taipei Medical University, Taipei, Taiwan; 9 Department of Neurosurgery, Taipei Medical University Hospital, Taipei, Taiwan; 10 Center for Neurotrauma and Neuroregeneration, Taipei Medical University, Taipei, Taiwan; 11 School of Public Health, College of Public Health and Nutrition, Taipei Medical University, Taipei, Taiwan; University of Michigan School of Medicine, UNITED STATES

## Abstract

**Background:**

High grade gliomas are the most common type of malignant brain tumor, and despite their rarity, cause significant morbidity and mortality. This study aimed to compare the treatment patterns of high grade glioma to examine survival patterns in patients who receive specific treatments between cohorts in Ohio and Taiwan.

**Method:**

Patients aged 18 years and older at age of diagnosis with World Health Organization (WHO) grade III or IV astrocytoma from 2007-2012 were selected from the Ohio Brain Tumor Study and the Taiwan Cancer Registry. The treatment information was derived from medical chart reviews in Ohio and National Health Insurance Research Data in Taiwan. Treatment examined included surgical procedure (brain biopsy and/or resection), radiotherapy (radiation and/or radiosurgery), and alkylating chemotherapy. Kaplan-Meier and parametric survival models were used to examine the effect of treatment on survival, adjusted for age, sex, and comorbidities.

**Results:**

294 patients in Ohio and 1,097 patients in Taiwan met the inclusion criteria. 70.3% patients in Ohio and 51.4% in Taiwan received surgical resection, followed by concurrent chemoradiation. Patients who received this treatment had the highest survival rate, with a 1-year survival rate of 72.8% in Ohio and 73.4% in Taiwan. Patients who did not receive surgical resection, followed by concurrent chemoradiation had an increased risk of death (hazard ratio of 5.03 [95% confidence interval (CI): 3.61-7.02] in Ohio and 1.49 [95% CI: 1.31-1.71] in Taiwan) after adjustment for age, sex, and comorbidities.

**Conclusion:**

Surgical resection followed by concurrent chemoradiation was associated with higher survival rate of patients with high grade glioma in both Ohio and Taiwan; however, one-third of patients in Ohio and half in Taiwan did not receive this treatment.

## Introduction

High grade gliomas, World Health Organization (WHO) grade III and IV, are a rare disease with very poor prognosis [1]. The incidence of high grade glioma is 3.56 per 100,000 population in the United States (3.19 per 100,000 for glioblastoma and 0.37 per 100,000 for anaplastic astrocytoma) [2], and 1.04 per 100,000 in Taiwan (L.N. Chien, unpublished data, manuscript under review). Although high grade glioma is often treatment resistant, therapeutic options have continued to expand, and some improvement has been seen in overall survival [3].

Age at diagnosis, Karnofsky performance status (KPS), histological type, and extent of surgical resection are the most significant validated prognostic factors in predicting overall survival of patients with high grade glioma [4–9]. Although many of these factors are not modifiable, extent of resection and chemotherapy have been shown to be associated with better survival for patients with high grade glioma [3, 7, 9–11] and these practices can be modified.

The standard treatment of high grade glioma has been reported from several clinical trials [12–14]; however, very few population-based studies have reported whether the treatment pattern was consistent with the recommended guidelines [15]. Understanding the treatment pattern and survival of high grade gliomas is crucial to better understand how treatment can improve the lives of patients in the real world. In this study, we used two population-based cohorts from the Ohio Brain Tumor Study and Taiwan to investigate the treatment pattern of the two most common high grade malignant gliomas, including glioblastoma (GB) and anaplastic astrocytoma (AA), and also to examine how the treatment pattern is associated with survival. Additionally, this international comparison study provides a novel analysis of the treatment patterns for high grade gliomas in two different geographic regions (North America versus East Asia), as well as different ethnic populations (European ancestry versus East Asian ancestry).

## Materials and Methods

### Ethics Statement

This study was approved by the Taipei Medical University Joint Institutional Review Board (approval no. 201402018), and the University Hospitals Case Medical Center Institutional Review Board (IRB number: CC296).

### Data sources

In Ohio, newly diagnosed untreated GB patients were prospectively recruited at the Brain Tumor and Neuro-Oncology Center at University Hospitals Case Medical Center (UHCMC), the clinical affiliate of the Case Western Reserve University (CWRU) School of Medicine, the Rose Ella Burkhardt Brain Tumor and Neuro-Oncology Center at the Cleveland Clinic (CC), the Dardinger Neuro-Oncology Center, Department of Neurosurgery at the James Cancer Hospital, and the Ohio State University Medical Center (OSU) and the Brain Tumor Center at the University of Cincinnati (UC), under the Ohio Brain Tumor Study (OBTS) Institutional Review Board (IRB) approved protocol [16]. All newly diagnosed primary brain tumor patients were identified at each center by research nurses, and written informed consent was obtained for all patients in the Ohio Brain Tumor Study. Clinical variables, such as comorbidities, final pathological diagnosis, and KPS, were abstracted from patient medical records. The patients used in this analysis represent approximately 11% of GB and AA patients diagnosed in Ohio during this time period (Central Brain Tumor Registry of the United States, unpublished data). All patients were followed actively until deaths or loss to follow-up (mean follow up months: 19.9).

The Taiwan Cancer Registry (TCR), a population-based cancer registry [17], was used to identify patients with GB and AA in Taiwan. Treatment information was derived from insurance claims of National Health Insurance Research Datasets (NHIRD), which contains data regarding nearly all of the clinical diagnoses, treatments, and prescription drugs that patients received. The NHIRD is maintained by the National Health Insurance Administration (NHIA) and aggregates reimbursement claims of beneficiaries enrolled in the National Health Insurance (NHI) program. Every citizen of Taiwan has been required by law to enroll in the NHI since 1995, and the coverage rate under NHI was 99% in 2012 [18]. Death records were obtained from the National Death Registry (NDR), a population-based registry for the cause of death in Taiwan. The completeness and accuracy of death records of Taiwan is high since it is mandatory to register all death with the NDR [19].

### Patient selection

We selected patients who were diagnosed with GB (International Classification of Diseases for Oncology, 3rd Edition [ICD-O-3]: 9440/3, 9441/3 and 9442/3) or AA (ICD-O-3: 9401/3) from the years of 2007 to 2012 in Ohio and from 2007 to 2010 in Taiwan, due to data availability. Patients who were less than 18 years old at age of diagnosis were excluded. Those with more than one primary cancer site were excluded from the Taiwan data, as it was difficult to identify their treatment based on insurance claims. A total of 294 patients in Ohio and 1,097 patients in Taiwan met the inclusion criteria.

### Treatment

The standard therapy for newly diagnosed malignant GB is surgical resection followed by concurrent chemoradiation where the chemotherapeutic drug of choice is temozolomide [3, 12–14]. Patients may also receive radiosurgery, or other first or second line chemotherapeutic agents. Current drugs used as an adjunct or alternate to chemotherapeutic treatment with temozolomide in the first and/or second line setting include: bevacizumab, carboplatin, cyclophosphamide, carmustine, etoposide, and Gliadel wafers. Some of these drugs are currently being investigated in clinical trials. Not all of these additional drugs are covered by Taiwan’s NHI or available in Taiwan. In the population of newly diagnosed grade III glioma with 1p/19q codeletion, the prevailing treatment recommendation is currently radiation therapy plus alkylating chemotherapy (temozolomide, or a combination of procarbazine, CCNU, and vincristine [PCV]). For grade III glioma patients without 1p /19q c-deletion, the prevailing recommendation is radiation therapy or chemotherapy alone as no benefit has been observed with chemotherapy concurrent to radiation therapy [20].

We classified chemotherapeutic treatment into temozolomide, bevacizumab, and other agents in the analysis. The treatment patterns were classified into six categories, including surgical resection followed by concurrent chemoradiation, surgical resection and radiation (with no chemotherapy), surgical resection (with no radiation or chemotherapy), concurrent chemoradiation (with no surgery other than biopsy), any other regimen, and no treatment. We additionally grouped patients into those that received surgical resection followed by concurrent chemoradiation and those that did not (referred to as all other treatments).

### Survival

The survival time was calculated using the date of death in medical records in Ohio and using the date of death from the NDR in Taiwan. Patients alive at the end of the follow up were censored. Mean follow-up in months was 19.9 for Ohio and 18.5 for Taiwan.

### Covariates

Covariates used in this analysis included age, sex, histological type, and comorbidities. Age at diagnosis was classified into 18–39, 40–64, and 65+ years. Comorbidities were computed based on the Charlson comorbidity index (CCI), which is regularly used in many claim-based cancer studies, to adjust for the effect of comorbidities on overall survival [21–24]. For Taiwan, a specific disease was defined if a patient had at least one diagnosis during hospital inpatient admission and two diagnoses in the outpatient visit one year prior to and one month after being diagnosed with cancer [25]. Since the information for Ohio patients was from medical records, we treated patients with a specific disease if the disease was recorded in their medical record. The CCI score of comorbidities was grouped into 0, 1–2, and 3+.

### Statistical analysis

Chi-square tests and Student’s t tests were used to compare the differences of patients’ characteristics between Ohio and Taiwan. Kaplan-Meier curves were used to describe the survival by histology. Since the models failed to pass the proportional hazards assumption, we alternatively used parametric survival analysis with a Weibull distribution to examine factors associated with treatment patterns. Statistical analyses were conducted using SAS/STAT, Version 9.3, STATA 12 (StataCorp LP, College Station, TX, USA), and R Version 3.1.1 software packages.

## Results and Discussion

### Basic demographics

Table 1 presents the basic characteristics of patients diagnosed with high grade glioma in Ohio and Taiwan. The mean age of the patients in Ohio was 60.6 years (standard deviation [SD]: 13.7) which was higher than that in Taiwan (mean: 58.4 years and SD of 15.9) (p = 0.019). A total of 62.2% of the patients in Ohio were male, and this was not significantly different from the patients in Taiwan (p = 0.324). Most of the patients in both sites were between the ages of 40 and 64 years at time of diagnosis and had no other comorbidities. Distribution of histologies significantly differed between the two sites: Ohio had more patients with GB (89.5%) than Taiwan (82.8%) (p = 0.005). For treatment, 88.1% of patients received surgical resection, 78.6% received radiation, and 72.8% received chemotherapy in Ohio, which was significantly higher than in Taiwan (p <0.001 for all treatments). Temozolomide was the most commonly used first line drug agent in Ohio (71.8%), followed by bevacizumab in the progression setting (24.8%); however, no patients were treated with bevacizumab in Taiwan since the drug was only approved to use when temozolomide failed to decrease the progression in their disease.

**Table 1:** Characteristics of Patients Diagnosed with the High Grade Glioma in Ohio and Taiwan.

			Ohio		Taiwan		
Variables			N	(%)	N	(%)	P value
Basic characteristics			294		1097		
Age in years, mean (SD)			60.6	(13.7)	58.4	(15.9)	0.019
Sex, male(%)			183	(62.2)	648	(59.1)	0.324
Age group	18–39		24	(8.2)	153	(13.9)	0.030
	40–64		149	(50.7)	518	(47.2)	
	65+		121	(41.2)	426	(38.8)	
CCI	0 score		183	(62.2)	617	(56.2)	<0.001
	1–2 score		61	(20.7)	384	(35.0)	
	3+ score		16	(5.4)	96	(8.8)	
	Missing medical record review		34	(11.6)	0	(0.0)	
Histology	GB		263	(89.5)	908	(82.8)	0.005
	AA		31	(10.5)	189	(17.2)	
Follow up months, mean (SD)			19.9	(13.3)	18.5	(15.4)	0.010
Initial treatment	Surgical resection		259	(88.1)	786	(71.6)	<0.001
	Radiation		231	(78.6)	828	(75.5)	<0.001
	Chemotherapy		214	(72.8)	755	(68.8)	<0.001
		Temozolomide	211	(71.8)	751	(68.5)	<0.001
		bevacizumab	73	(24.8)	0	(0.0)	<0.001
		All others	57	(19.4)	6	(0.5)	<0.001
	Missing treatment information		28	(9.5)	0	(0.0)	<0.001

GB: glioblastoma; AA: anaplastic astrocytoma; CCI: Charlson comorbidity index; SD: standard deviation

### Survival and treatment pattern

**Fig 1:**
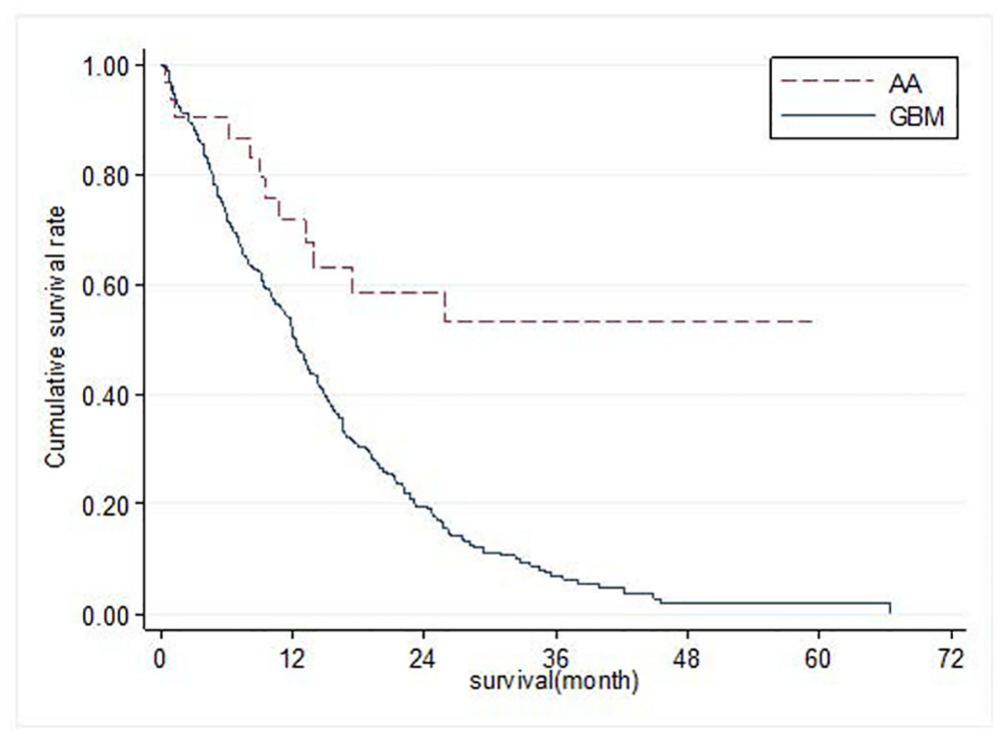
Overall Survival of Patients with Anaplastic Astroctyoma (AA) or Glioblastoma (GBM) in Ohio.

**Fig 2:**
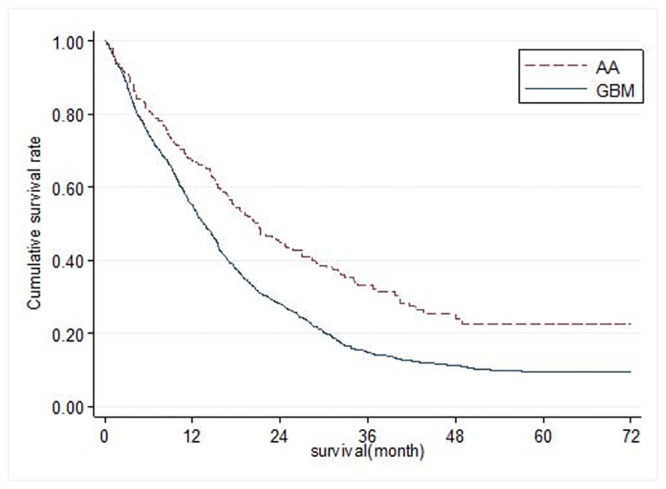
Overall Survival of Patients with Anaplastic Astroctyoma (AA) or Glioblastoma (GBM) in Taiwan.

Overall survival is shown in Figs 1 and 2. The survival rate was lower in patients with GB than those with AA in both Ohio and Taiwan. The 1-year survival of GB was about 50% in both sites (Ohio: 51.6%, Taiwan: 55.0%) while 1-year survival of AA in Ohio and in Taiwan was approximately 70% for both (Ohio: 69.2%, Taiwan: 67.2%). The 2-year survival of GB was 17.2% in Ohio, which was lower than that in Taiwan (28.1%). The 2-year survival of AA was 52.2% in Ohio and 45.0% in Taiwan.

Further investigation of the treatment pattern of patients showed that 70.3% in Ohio and 51.4% in Taiwan received surgical resection followed by concurrent chemoradiation. The second most common treatment combination was surgical resection alone (with no radiation or chemotherapy) in Ohio. In Taiwan, the second most common pattern was concurrent chemoradiation with no resection, (11.8%) while this was observed in only 6.8% of patients in Ohio. Other treatment patterns that were observed included: surgical resection followed by radiation (6.8% in Ohio and 7.9% in Taiwan), and treatment with other agents (3.8% in Ohio and 10% in Taiwan). A small proportion—1.5% in Ohio, and 9.7% in Taiwan—of patients received no treatment. In terms of survival rate, patients who received surgical resection followed by concurrent chemoradiation had the highest survival rates in both sites, with a 1-year survival rate of 72.8% in Ohio and 73.4% in Taiwan. Patients with concurrent chemoradiation also had relatively better survival, with a 1-year survival rate of 48.2% in Ohio and 69.0% in Taiwan. For those with surgical resection only or no treatment, the survival rate was lowest in both sites (Table 2).

**Table 2:** Survival Rates stratified by treatment pattern for Patients Diagnosed with the High Grade Glioma in Ohio and Taiwan.

		Ohio						Taiwan					
		N	(%)	1-year survival		2-year survival		N	(%)	1-year survival		2-year survival	
Treatment pattern				Rate	(95% CI)	Rate	(95% CI)			Rate	(95% CI)	Rate	(95% CI)
Overall		294	(100)	54.0	(48.8–60.1)	23.0	(19.3–30.1)	1097	(100)	56.5	(53.5–59.4)	30.6	(27.9–33.4)
Surgical Resection + concurrent chemoradiation		187	(70.3)	72.8	(67.0–80.0)	33.2	(27.0–41.0)	564	(70.3)	73.4	(69.6–76.9)	38.1	(34.1–42.1)
	All other treatments	79	(29.7)	21.5	(14.0–33.0)	3.5	(1.0–13.0)	533	(29.7)	38.7	(34.5–42.8)	22.7	(19.2–26.3)
	Surgical Resection + Radiation	18	(6.8)	11.1	(3.0–41.0)	0.0		87	(6.8)	56.3	(45.3–66.0)	34.5	(24.7–44.4)
	Surgical Resection only	29	(10.9)	3.6	(1.0–25.0)	0.0		101	(10.9)	8.9	(4.4–15.4)	6.9	(3.1–13.0)
	Concurrent chemoradiation	18	(6.8)	48.2	(29.0–79.0)	13.8	(4.0–49.0)	129	(6.8)	69.0	(60.2–76.2)	42.6	(34.0–51.0)
	Others	10	(3.8)	50.0	(27.0–93.0)	0.0		110	(3.8)	38.2	(29.2–47.1)	19.1	(12.4–26.9)
	No treatment	4	(1.5)	0.0		0.0		106	(1.5)	16.0	(9.8–23.6)	7.6	(3.5–13.6)

CI: confidence interval

### Regression analysis

Table 3 presents the hazard ratios of death among patients with high grade glioma. Increased risk of death in the Ohio cohort was associated with older age, male sex, diagnosis of GB, and not receiving surgical resection followed by concurrent chemoradiation. The same pattern was found in Taiwan. The treatment was further categorized into surgical resection followed by concurrent chemoradiation and all other treatments, the risk of death of the patients who received all other treatments was higher than those that received surgical resection followed by concurrent chemoradiation in Ohio (hazard ratio (HR) = 5.03, 95% confidence interval (CI): 3.61–7.02) and in Taiwan (HR = 1.49, 95% CI: 1.31–1.71).

**Table 3:** Multivariable Survival Models of the Hazard Risk of Death of Patients Diagnosed with the High Grade Glioma for Ohio and Taiwan.

	Model 1						Model 2					
	Ohio			Taiwan			Ohio			Taiwan		
Variables	HR	(95% CI)	*P*	HR	(95% CI)	*P*	HR	(95% CI)	*P*	HR	(95% CI)	*P*
Age group (Ref. = 18–39)	1.00	(Ref.)		1.00	(Ref.)		1.00	(Ref.)		1.00	(Ref.)	
40–64	1.97	(0.78–4.94)	0.152	1.28	(1.02–1.6)	0.031	1.77	(0.72–4.46)	0.224	1.30	(1.04–1.62)	0.023
65+	3.51	(1.36–9.03)	0.010	2.35	(1.85–2.99)	< 0.001	3.99	(1.56–10.19)	0.004	2.81	(2.22–3.56)	< 0.001
Sex (Ref. = Male)	1.00	(Ref.)		1.00	(Ref.)		1.00	(Ref.)		1.00	(Ref.)	
Female	0.69	(0.51–0.93)	0.014	0.83	(0.72–0.95)	0.006	0.72	(0.54–0.97)	0.030	0.86	(0.75–0.98)	0.025
Histological type (Ref. = GB)	1.00	(Ref.)		1.00	(Ref.)		1.00	(Ref.)		1.00	(Ref.)	
AA	0.33	(0.17–0.64)	0.001	0.71	(0.58–0.86)	< 0.001	0.33	(0.17–0.64)	0.001	0.67	(0.55–0.81)	<0.001
CCI (Ref. = No comorbidity)	1.00	(Ref.)		1.00	(Ref.)		1.00	(Ref.)		1.00	(Ref.)	
1–2 score	0.92	(0.65–1.30)	0.631	1.02	(0.88–1.18)	0.773	0.98	(0.70–1.36)	0.883	1.00	(0.87–1.16)	0.969
3+ score	1.58	(0.92–2.74)	0.100	1.17	(0.93–1.48)	0.184	1.43	(0.84–2.44)	0.189	1.26	(1–1.59)	0.049
Treatment (Ref. = surgical resection followed by concurrent chemoradiation)	1.00	(Ref.)		1.00	(Ref.)		1.00	(Ref.)		1.00	(Ref.)	
Surgical resection + Radiation	7.04	(4.00–12.4)	< 0.001	1.07	(0.82–1.4)	0.606						
Surgical resection only	11.8	(7.17–19.4)	< 0.001	3.22	(2.55–4.05)	< 0.001						
Concurrent chemoradiation	2.59	(1.44–4.67)	0.001	0.86	(0.69–1.08)	0.197						
Others	3.21	(1.55–6.68)	0.001	1.57	(1.25–1.97)	< 0.001						
No treatment	20.6	(7.07–60.0)	<0.001	2.97	(2.38–3.71)	<0.001						
All other treatment							5.03	(3.61–7.02)	< 0.001	1.49	(1.31–1.71)	< 0.001

Model 1 used the detail treatment category; Model 2 used surgical resection followed by concurrent chemoradiation versus all the others.

GB: glioblastoma; AA: anaplastic astrocytoma; CCI: Charlson comorbidity index; HR: hazard ratio; CI: confidence interval

### Median survival time for GB

Overall, the adjusted median survival time for patients with GB was 14.2 months (95% CI: 13.4–15.0) in Ohio and 15.1 months (95% CI: 14.8–15.4) in Taiwan. Of the patients who received surgical resection followed by concurrent chemoradiation, the median survival time increased by approximately 10 months in Ohio as well as in Taiwan as compared to the patients with all other treatments. The adjusted median survival time of the patients who received concurrent chemoradiation alone was the second highest group in Taiwan (22.7 months, 95% CI: 21.1–24.3), which was 1.9 months higher than the patients who received surgical resection followed by concurrent chemoradiation (Table 4).

**Table 4:** Median Survival Time (Months) of Patients with Glioblastoma in Ohio and Taiwan.

		Ohio					Taiwan				
Treatment pattern		N (%)	Crude	(95% CI)	Adjusted*	(95% CI)	N (%)	Crude	(95% CI)	Adjusted*	(95% CI)
Total		263(100.0)	12.1	(10.9–14.2)	14.2	(13.4–15.0)	908(100.0)	13.5	(12.6–14.8)	15.1	(14.8–15.4)
Surgical Resection + concurrent chemoradiation		167(63.0)	16.6	(14.9–19.3)	19.1	(18.5–19.6)	487(53.6)	18.3	(16.5–19.6)	20.8	(20.4–21.2)
	All other treatments	71(27.0)	4.5	(3.9–6.6)	8.9	(8.1–9.8)	421(46.4)	7.2	(6.0–8.3)	9.7	(9.3–10.1)
	Surgical Resection + Radiation	17(6.5)	4.5	(3.7–7.1)	6.8	(6.5–7.1)	53(5.8)	10.3	(6.4–15.7)	15.5	(12.2–18.7)
	Surgical Resection only	27(10.5)	3.3	(1.6–4.6)	3.4	(3.2–3.6)	84(9.3)	3.5	(2.7–3.9)	4.1	(3.7–4.5)
	Concurrent chemoradiation	16(6.1)	10.8	(4.4–19.2)	17	(14.4–19.6)	102(11.2)	16.7	(14.6–22.5)	22.7	(21.1–24.3)
	Others	9(3.4)		--	--	--	90(9.9)	8	(6.4–10.4)	11.1	(9.8–12.3)
	No treatment	2(0.8)		--	--	--	92(10.1)	3.2	(2.8–4.4)	4.6	(4.2–5.1)

*Adjusted by age, sex and comorbidities

-- excluded due to low numbers

CI: confidence interval

This study provides a unique opportunity to describe the current treatment patterns of adult patients with high grade glioma in two different populations. Patients were prospectively enrolled in Ohio but retrospectively studied in the Taiwan cohort derived from Taiwan Cancer Registry and the periods of enrollment slightly differed between Ohio and Taiwan. At the time of this analysis, data was only available until the year 2010 for the TCR. High grade gliomas—in particular, grade III gliomas—are a rare disease, and it may take an extended period of time to recruit a sufficiently large cohort for an analysis within a network. As a result, a longer period of recruitment was allowed for the Ohio cohort, in order to significantly increase the number of patients included in the analysis.

The treatment pattern between the two sites significantly differed. In Ohio, the majority of patients received surgical resection followed by concurrent chemoradiation, while in Taiwan only half of patients received this regimen. The largest difference between two regions was whether patients received any surgical resection (88% in Ohio and 72% in Taiwan), as compared to receiving chemoradiation without resection. Those that received surgical resection followed by concurrent chemoradiation had highest survival in both sites after adjustment for age at diagnosis, sex, and comorbidities. The 1-year survival rate of the patients with surgical resection followed by concurrent chemoradiation was around 73% in both sites. In addition, the adjusted median survival time was 10 months higher in these patients as compared to those patients who received all other treatments in Ohio as well as in Taiwan.

When we limited our data to the patients who received either biopsy or resection, around 80% of patients had chemotherapy and radiation in Ohio as well as in Taiwan. This is higher than another analysis of a commercially insured US population where of the 2,272 patients that underwent resection for high grade glioma, 37.0% received temozolomide and radiation therapy, 13.8% received radiation alone, 3.9% received temozolomide alone, and 45.3% of patients received neither [15]. Other studies have also reported that use of chemotherapy for treatment of high grade glioma may be lower than would be expected given published treatment recommendations [26]. This difference could potentially have several different explanations. In Ohio, patients were recruited from tertiary referral medical centers. Patients at these institutions may have access to clinical trial protocols, and physicians at these institutions may be more likely to pursue more aggressive therapy and follow published treatment guidelines. Patients that seek treatment at these institutions may be more likely to have health insurance coverage, or ability to self-pay for aggressive treatment. As the Taiwanese sample covers the entire country, there should be no effect related to type of institution. Additionally, there is universal health insurance in Taiwan, and there would be few differences in utilization of aggressive forms of treatment due to ability to pay.

It was expected that the patients in Taiwan might receive more surgical procedures since the mean age of these patients was younger than that in Ohio (71.6% in Taiwan versus 88.1% in Ohio). One possible explanation is that the patients in Ohio were recruited from the four major academic medical centers in the state and thus may have had access to better health care resources and clinical trials, whereas patients in Taiwan were drawn from the entire population. Additionally, the more conservative treatment pattern in Taiwan might demonstrate a cultural difference between the West and the East, similar to that previously shown in a study of breast cancer [27]. Approximately 10% of the patients in Taiwan had no treatment, which could be because Taiwan’s NHI program covers traditional Chinese medicine, and this population may represent patients who chose to seek this alternative treatment.

Treatment pattern can be affected by many factors, including current therapeutic trends, patient characteristics (including demographics, comorbidities, perceived severity of tumor, and patient preference among others), physician characteristics, available health care resources, and insurance coverage. Therapeutic policy may also be changed depending on pathological types, contemporary tendency of management and other unmeasured factors. However, we were unable to get more homogenous data from both sites to provide better interpretation of our finding. Besides, in certain circumstances, the recommended standard of treatment may not be the best option for a patient. The elderly (especially those over 70) [28], and those with low performance status (KPS< 70) with grade IV glioma are particularly likely to not receive surgical resection followed by concurrent chemoradiation, and are usually excluded from clinical trials for high grade glioma in the United States. However, this issue was limited to a very small number of patients and should not have influenced the results.

More aggressive treatment was associated with better survival in this study, which is consistent with previous studies. Several previous studies have shown that median survival among patients who received partial or total resection is significantly higher than those who receive biopsy only [4], and that there is a significant improvement in overall survival with resection followed by concurrent chemoradiation [29]. In this study, we found patients with GB who received surgical resection followed by concurrent chemoradiation had the highest survival, surviving 10 months longer than those who received all other treatments, after adjusting age, gender, and comorbidities in Ohio. This may be biased by the fact that patients who survive longer after diagnosis are more likely to receive aggressive treatment. When we limited analysis just to those patients that survived 3 months or more, we found a 7% increase in the amount of patients that received surgical resection followed by concurrent chemoradiation in both Ohio and Taiwan.

Temozolomide is still the most common chemotherapeutic agent in this study, which follows the prevailing treatment recommendations for high grade glioma [30]. Though bevacizumab is not a part of the first line standard treatment regimen for high grade glioma, a substantial proportion of patients in Ohio also received this drug. The amount of patients treated with this drug reached a maximum with the group diagnosed in 2012 where 50% of GB patients received this drug. As these patients all received treatment at academic medical centers, this may also represent a portion of patients that participate in clinical trials. Usage of chemotherapy is highly regulated by the NHI in Taiwan, which significantly limits the types of drugs that this patient population can receive. Bevacizumab is also reimbursed in Taiwan’s NHI program but its use is limited only to patients who have had tumor progression while receiving temozolomide. Recent randomized controlled trials have not shown significant improvement for patients diagnosed with GB who used this chemotherapeutic agent [31, 32]. Whether this finding affects the current practice, future research is needed to further investigate.

In agreement with other studies, we also found that younger age and female gender were associated with better survival. Previous studies have found an association between long-term survival after diagnosis with glioblastoma and young age at diagnosis and high post-surgical KPS (score of 70 or greater) [5, 8]. We were unable to control for factors that have been strongly associated with overall survival, such as mental status, as these variables were not consistently available patient medical records in both sites. 38.1% of Ohio patients were missing KPS data, so we were unable to adjust for the potential effect of KPS in this study. Though CCI has been found to be a significant covariate in other claim-based studies, we did not find CCI score to be significantly associated with survival in this study. The quality of initial resection can also affect the survival but cannot be addressed in this study due to limitations in the data used for study.

Research in cancer cell lines and mice has demonstrated that blocking critical DNA repair mechanisms could improve the effectiveness of radiation therapy for GB. Radiation therapy causes double-strand breaks in DNA that must be repaired for tumors to keep growing [33–35]. This study found that patients who received radiation in Taiwan had better survival than those in the United States, which might suggest that patients in Taiwan had better response to radiation therapy. This might also suggest genetic differences in DNA repair mechanisms or other critical treatment response pathways between those of European ancestry and those of Asian ancestry. Asians may have specific polymorphisms in specific treatment response related genes that lead to better response to radiotherapy. Some molecular markers that have been shown to mediate response to chemotherapy and radiation—in particular isocitrate dehydrogenase 1/2 (*IDH1/IDH2*) mutation—are more common AA than in GB [36]. This molecular information was not consistently available for the patients included in the analysis, but variation in these factors may also have had an influence on the results of this analysis.

This study used an analogous approach comparing the treatment pattern and survival of high grade glioma in two different populations with the reliability and comparability of the available data. The results were subjected to some limitations. The distribution of GB and AA differed between the 2 cohorts (10.5% [N = 31] in Ohio and 17.2% [N = 189] in Taiwan). This was seen in comparing the incidence of malignant brain and CNS tumor in the US with that in Taiwan. The incidence of GB and AA in the US were both significant higher than that in Taiwan; however, the proportion of AA in all tumors was higher in Taiwan (7.6%) than that in the US (5.5%)(L.N. Chien, unpublished data, manuscript under review). Unlike Taiwan, the United States does not have a national health insurance system so population-based treatment information is not available. The patient population from Ohio was recruited from academic medical centers that have substantial resources and as a result is not representative of the United States as a whole. As compared to the national population of GB and AA patients, the OBTS population is slightly younger, and more male [2]. Besides, treatment information for the Taiwanese population was abstracted from insurance claims; thus, we were unable to observe the treatment that may have been paid for through alternate means. Thus, treatment patterns may be significantly affected by NHI policy.

AA is much less common than GB within both populations. The proportion of Ohio patients with AA is low, and this may make it difficult to detect the true survival differences that may exist between this group and the Taiwanese population. Treatment protocols for AA are less established than those for GB. Different institutions may have different standard patterns of treatment for AA, as opposed to GB where there is an evidence-based standard treatment (surgical resection followed by concurrent chemoradiation). One recent analysis found that those who received radiation treatment alone for AA had improved overall survival as compared to persons that received concurrent chemoradiation or radiation followed by chemotherapy [37]. As a result, it is possible that this analysis did not use the appropriate treatment groupings necessary in order to elucidate the survival patterns after diagnosis with AA in these two populations.

## Conclusions

In conclusion, this study found that treatment patterns for high grade glioma differ between Ohio and Taiwan. The results might reveal the difference in clinical practice, patient’s preference, as well as the therapy response. However, patients who received surgical resection followed by concurrent chemoradiation had better survival compared to all the others in both sites. For the patients who received no treatment beyond biopsy, the survival rate was very low. Future research is critical to better understand the reasons for these treatment patterns in these patient populations.
